# Evaluating the Therapeutic Role of Lymph Node Dissection in Variant Subtype Bladder Cancer

**DOI:** 10.3390/cancers17152536

**Published:** 2025-07-31

**Authors:** Syed Nahiyaan Rahman, Darryl T. Martin, Kandala Keervani, Spencer James, Peter Humphrey, David Hesse, Wei Shen Tan, Sunil Patel, Jonathan Wright, Fady Ghali

**Affiliations:** 1 Department of Urology, Yale University School of Medicine, New Haven, CT 06520, USA; syed.rahman@yale.edu (S.N.R.); kandala.keervani@yale.edu (K.K.); david.hesse@yale.edu (D.H.); weishen.tan@yale.edu (W.S.T.); 2 Department of Urology, University of Washington School of Medicine, Seattle, WA 98195, USA; spencj@gmail.com; 3 Department of Pathology, Yale University School of Medicine, New Haven, CT 06520, USA; peter.humphrey@yale.edu (P.H.);; 4 James Buchanan Brady Urological Institute, Johns Hopkins Medicine, Baltimore, MD 21201, USA; spate167@jhmi.edu

**Keywords:** node dissection, variant, cystectomy, urothelial

## Abstract

Background: The importance of lymph node dissection (LND) at the time of radical cystectomy for urothelial carcinoma (UC) is widely accepted despite known risks. The therapeutic benefits of LND for variant subtype bladder cancer (VBC), a heterogenous and distinct set of diseases, are not well established. We aim to characterize the impact of LND on overall survival across VBC subtypes. Methods: The National Cancer Database was queried for all cases of variant subtype bladder cancer managed with radical cystectomy between 2004 and 2020, using the International Classification of Disease-O-3 morphological codes. The cases were stratified by receipt of individual variant subtypes. The primary outcome was overall survival associated with pathologic nodal status and receipt of nodal dissection. A Kaplan–Meier analysis and Cox proportional hazards analysis were used for survival analyses. Results: A total of 30,911 patients with VBC that were managed with radical cystectomy were included in our analysis. The pNx rates ranged from 33.1% in the micropapillary subtype, 42.2% in the sarcomatoid subtype, 68.4% in the squamous subtype, 48.9% in the adenocarcinoma subtype, and 56.2% in the neuroendocrine subtype. The median OS was higher in those that received a nodal dissection across subtypes but was statistically significant only for the squamous (71.0 [68.0 vs. 74.0] vs. 37.2 [33.6 vs. 40.9] months *p* < 0.001) and adenocarcinoma (45.9 [32.9 vs. 59.0] vs. 37.9 [28.6 vs. 47.1] months *p* = 0.037) subtypes. Using Cox proportional hazards regression, LN dissection was associated with improved OS for the squamous (0.50 (0.44–0.58) *p* < 0.001) and adenocarcinoma (0.65 [0.45–0.93) *p* = 0.030) subtypes. Conclusions: The role of LND across VBC subtypes is not clearly defined and warrants further investigation to develop a more risk-adaptive approach. We demonstrate heterogeneity with respect to the OS benefit associated with LND at the time of surgery. Among certain VBC subtypes, LND may not offer a significant therapeutic benefit, while LND in squamous and adenocarcinoma VBCs is correlated with improved survival.

## Simple Summary

The impact of lymph node dissection at the time of radical cystectomy for urothelial carcinoma has not been well established in the context of variant histologies. The aim of our study was to characterize the impact of node dissection on overall survival across various variant subtypes. We report significant heterogeneity in the use and benefit of LND, with squamous and adenocarcinoma subtypes showing the clearest survival benefit. These findings highlight the importance of a more risk-adapted surgical approach in VBC and the need for further investigation of subtype-specific therapeutic strategies.

## 1. Introduction

Surgical extirpation is the backbone therapy for localized bladder cancer (BC), yet the inclusion and extent of lymph node dissection (LND) at the time of radical cystectomy (RC) remains an area of active investigation. Several studies have explored the impact of the performance and extent of LND in urothelial carcinoma (UC), the most common subtype of BC, and support LND at the time of surgery [1,2]. Still, recent prospective randomized data have suggested that extended LND does not improve outcomes, and indeed is associated with increased mortality, in UC when compared with more limited removals of nodes [3].

Variant subtype BC (VBC) is a heterogenous group of diseases that are harbored in almost 5% of all bladder cancer cases [4]. This group is biologically distinct from UC in important ways. The careful study of the clinical behavior of VBC has demonstrated atypical patterns of spread, varying aggressiveness, chemo-sensitivity, and immune-sensitivity [5,6,7,8]. The impact of LND and lymph node yield (LNY) at the time of RC in VBC remains under-investigated. Given the evidence of significant increases in morbidity with extensive nodal dissection, careful consideration of the role of LND in VBC is warranted [3,9].

We sought to evaluate the impact of LND and LNY at the time of RC on overall survival (OS) in VBC. We hypothesize that there will be significant heterogeneity in the impact of LND and LNY on the outcomes for various histological subtypes, and thus, may inform more individualized surgical decision-making rooted in disease biology.

## 2. Methods

### 2.1. Data Source

The National Cancer Database (NCDB) includes approximately 70% of all new cancer diagnoses annually from over 1500 programs participating in the American College of Surgeons Commission. We queried bladder cancer cases in the NCDB from 2004 to 2020 to identify histologic urinary bladder cancer (International Classification of Diseases for Oncology, 3rd Edition topography codes C67.0–C67.9). We identified patients with variant subtype UC including micropapillary, sarcomatoid, squamous, adenocarcinoma, neuroendocrine, and plasmacytoid subtypes based on the codes 8131, 8122, 8070/8051, 8140, 8041/8261, and 8082. These codes do not represent mixed histologies and are pure variants. We excluded patients who were not managed by RC and those with incomplete data regarding chemotherapy and staging. The final analytic cohort consisted of 30,911 patients with VBC. We extracted clinical and demographic characteristics including age, sex, histology, receipt of neoadjuvant chemotherapy (NAC), clinical T/N staging, and pathologic T/N staging.

### 2.2. Study Outcomes

The primary endpoint was to identify if nodal dissection at the time of radical cystectomy was associated with a survival benefit across variant histological subtypes. The secondary endpoints included the association of pN status with overall survival, lymph node yield, and receipt of NAC with overall survival.

### 2.3. Statistical Analysis

We characterized clinical and demographic characteristics using descriptive statistics for each identified variant histological subtype. A Kaplan–Meier analysis was used across each histological subtype to assess the relationship between the performance of a lymph node dissection at the time of radical cystectomy, the pN status (included pN0, pN+, and pNx), the lymph node yield for those that did receive a nodal dissection with a 15-lymph node cut off, and overall survival. Additionally, we substratified histological subtypes by their receipt of NAC and performed Kaplan–Meier analyses to assess the relationship between the performance of a lymph node dissection at the time of radical cystectomy and the overall survival by receipt of NAC (given the population size, this was only feasible for the sarcomatoid, squamous, and adenocarcinoma subtypes).

Cox proportional hazards models were created for each subtype, incorporating clinical parameters such as the clinical T stage, NAC receipt, and the performance of a nodal dissection to assess the impact of nodal dissection and survival (this was unable to be performed for plasmacytoid given the lower sample size compared to the rest). These factors were chosen as they are taken into account when deciding to perform a node dissection at the time of radical cystectomy on patients with a variant subtype. All statistical tests were two-sided with statistical significance defined as *p* < 0.05. Statistical analyses were conducted using the SPSS Statistics software (version 25, International Business Machines Corporation, Armonk, NY, USA).

## 3. Results

Between 2004 and 2020, we identified 30,911 patients with VBC that was managed with radical cystectomy (Table 1). A total of 426 patients with the micropapillary subtype, 704 patients with the sarcomatoid subtype, 28,491 patients with the squamous subtype, 601 patients with the adenocarcinoma subtype, 614 patients with the neuroendocrine subtype, and 75 patients with the plasmacytoid subtype were included in our analysis. The pNx rates ranged between 33.1% in the micropapillary subtype, 42.2% in the sarcomatoid subtype, 68.4% in the squamous subtype, 48.9% in the adenocarcinoma subtype, 56.2% in the neuroendocrine subtype, and 22.7% in the plasmacytoid subtype.

### 3.1. pN Status

**Figure 1:**
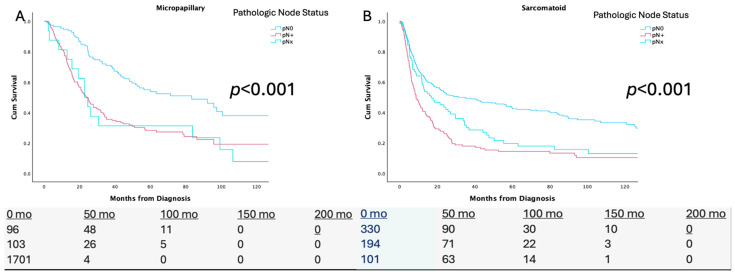

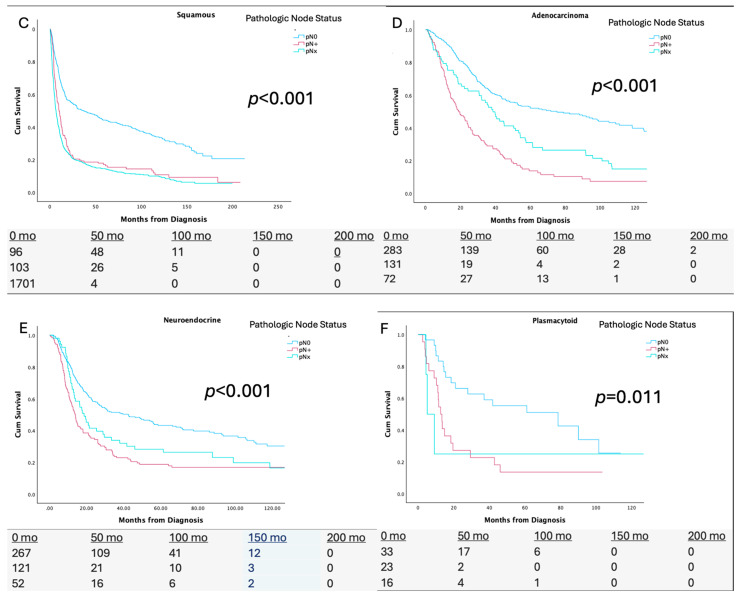
Kaplan–Meier Analysis stratified by pathologic nodal status (pN0/+/x) for (**A**) Micropapillary, (**B**) Sarcomatoid, (**C**) Squamous, **(D**) Adenocarcinoma, (**E**) Neuroendocrine, and (**F**) Plasmacytoid variant bladder cancer.

Figure 1 demonstrates the Kaplan–Meier analyses across the pN0/+/x status for each variant histological subtype. For the micropapillary subtype, the median OS ranged between 83.3 months for pN0, 23.9 months for pN+, and 22.7 months for pNx. For the sarcomatoid subtype, the median OS ranged between 31.1 months for pN0, 8.7 months for pN+, and 16.9 months for pNx. For the squamous subtype, the median OS ranged between 36.9 months for pN0, 10 months for pN+, and 6.5 months for pNx. For the adenocarcinoma subtype, the median OS ranged between 75.2 months for pN0, 19.0 months pN+, and 38.6 months for pNx. For the neuroendocrine subtype median OS ranged from 42.4 months for pN0, 13.7 months for pN+, and 18.6 months for pNx. For the plasmacytoid subtype, the median OS ranged between 78.6 months for pN0, 12.6 months for pN+, and 5 months for pNx.

### 3.2. Node Dissection and Nodal Yield

**Figure 2:**
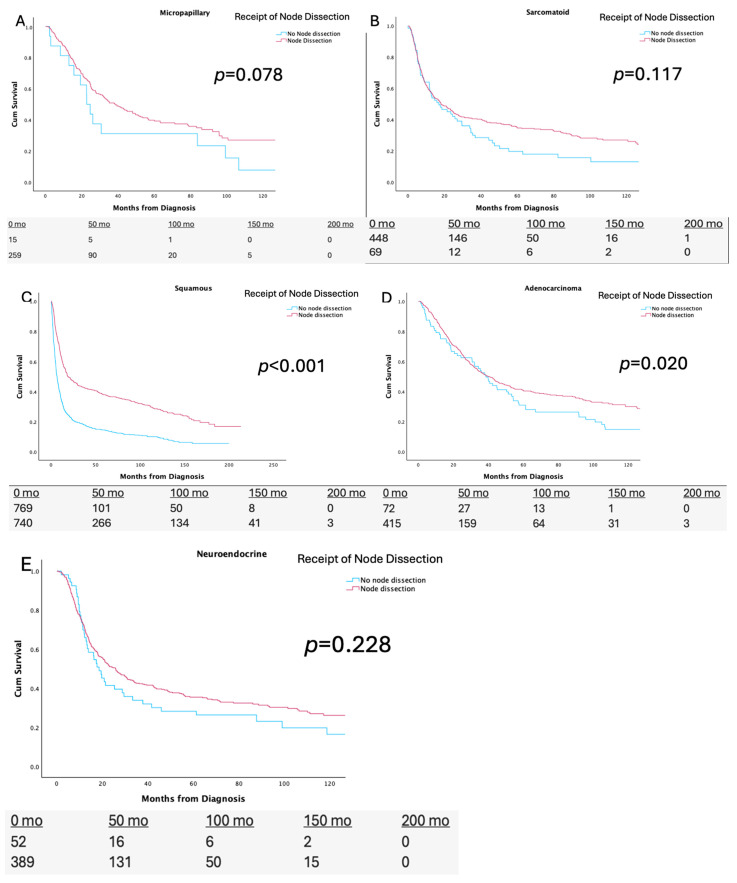
Kaplan–Meier analysis stratified by receipt of lymph node dissection (LND) for (**A**) Micropapillary, (**B**) Sarcomatoid, (**C**) Squamous, (**D**) Adenocarcinoma, and (**E**) Neuroendocrine variant bladder cancers.

Figure 2 demonstrates the Kaplan–Meier analyses, stratified by performance, of lymph node dissections at the time of radical cystectomy for each variant histological subtype. Among the squamous subtype, the median OS was statistically significantly higher for those that received a node dissection compared to those that did not (28.8 [14.1 vs. 36.6] vs. 6.5 [5.6 vs. 7.4] months *p* < 0.001). For the adenocarcinoma subtype, the median OS was statistically significantly higher in those that received a nodal dissection compared to those that did not (45.9 [32.9 vs. 59.0] vs. 37.9 [28.6 vs. 47.1] months *p* = 0.037). Among the micropapillary, neuroendocrine, and sarcomatoid subtypes, the median OS was not significantly higher in those that received a node dissection compared to those that did not (*p* > 0.05). The Kaplan–Meier analysis for the plasmacytoid subtype was excluded given its small sample size precluding quality parameters.

Supplementary Figure S1 demonstrates the Kaplan–Meier analyses, stratified by lymph node yield, for each variant histological subtype. For the micropapillary subtype, the median OS was significantly higher for those with a higher lymph node yield (51.6 vs. 32.0 months, *p* = 0.002). For the sarcomatoid subtype, the median OS did not significantly differ by nodal yield (23.5 vs. 17.9 months, *p* = 0.064). For the squamous subtype, the median OS was significantly higher for those with a higher nodal yield (37.3 vs. 8.1 months, *p* < 0.001). For the adenocarcinoma subtype, the median OS was significantly higher for those with a higher nodal yield (60.1 vs. 38.6 months, *p* = 0.019). For the neuroendocrine subtype, the median OS was significantly higher for patients with a higher LN yield (55.7 vs. 22.6 months, *p* = 0.005). For the plasmacytoid subtype, the median OS did not significantly differ by nodal yield (44.4 vs. 11.3 months, *p* = 0.090).

### 3.3. Multivariate Cox Proportional Hazards Analysis

Multivariate Cox Proportional Hazards models were created across all variants incorporating the clinical T stage, NAC receipt, receipt of LN dissection, and age (Table 2). Age was significantly associated with worse OS across all histologies (all *p* < 0.04), except plasmacytoid. NAC receipt was associated with improved OS for neuroendocrine (0.09 [0.07–0.94] *p* = 0.002) variant histologies. LN dissection was associated with improved OS for squamous (0.50 (0.44–0.58) *p* < 0.001) and adenocarcinoma (0.065 [0.045–0.93) *p* = 0.030) variant histologies. cN+ was associated with worse OS for squamous (1.66 [1.34–1.83] *p* = 0.03) and neuroendocrine histologies (1.34 [1.13–1.50] *p* = 0.02).

### 3.4. Receipt of Nodal Dissection Stratified by Receipt of NAC

We investigated the impact of the receipt of NAC on LND-associated benefits. NAC was received by 18.9% of the micropapillary, 13.3% of the sarcomatoid, 21.4% of the squamous, and 13.8% of the adenocarcinoma subtype. Patients with the squamous subtype observed a statistically significant survival benefit with LND, irrespective of the receipt of NAC (83.6 vs. 54.6, *p* < 0.001 and 98.4 vs. 75.9, *p* < 0.001 for NAC and no-NAC, respectively). Patients with the adenocarcinoma subtype that were NAC-naïve had an OS benefit associated with LND (NAC 83.7 months vs. 52.0 months, *p* = 0.041), which was attenuated by NAC exposure (70.3 months vs. 68.2 months, *p* = 0.341). Conversely, patients with the micropapillary and sarcomatoid subtypes did not have an LND-associated survival benefit in either the NAC or no-NAC subgroups (*p* > 0.05). These results can be seen in Supplementary Table S1.

## 4. Discussion

Lymph node dissection at the time of radical cystectomy is the standard of care for urothelial BC and is supported by retrospective studies in UC [1,2,10]. We evaluated the impact of LND amongst patients with VBC. Significant heterogeneity was observed with respect to the therapeutic impact correlated with the performance and extent of lymphadenectomy across various subtypes, though a strong prognostic benefit was seen across all groups.

The therapeutic benefit of LND at the time of cystectomy for UC is widely accepted amongst clinicians and is considered the standard of care (2) [11]. Clinical evidence suggests that the performance of LND and a higher LNY is associated with improved recurrence-free survival (RFS) and OS (2). Leissner et al., for example, reported a correlation between LNY and 5-year disease-free survival across pT1–3 patients with UC [12]. Nodal dissection resulting in greater than 15 lymph nodes was significantly associated with improved disease-free and cancer specific survival (*p* < 0.013 and *p* < 0.016, respectively) [12]. An analysis of the Surveillance, Epidemiology, and End Results database evaluated the effect of LND, stratified by stage, in UC and found the exclusion of LND was correlated higher overall mortality across all stages [13]. VBC, conversely, is a heterogenous group of diseases which warrants an independent investigation. To avoid the Will Rogers phenomenon in assessing LND benefits, we grouped positive and negative nodal statuses and compared any LND performed with no LND performed [14,15]. When evaluated separately, we found significant divergence with respect to any detectable benefit for histological variants.

Squamous BC, as an example, demonstrates clear survival benefits correlated with lymphadenectomy. Patients receiving LND have improved OS (28.8 [14.1 vs. 36.6] vs. 6.5 [5.6 vs. 7.4] months, *p* < 0.001 for LND vs. no LND) and a LNY of > 15 nodes, further demonstrating a positive correlation with improved OS (37.3 vs. 8.1 months, *p* < 0.001). An evaluation by pathologic nodal status among squamous BCs demonstrates that patients not receiving lymphadenectomy have survival rates akin to those with node positive diseases. This is further supported by observations in the multivariate regression model, where the squamous subtype demonstrates a significant association between LND and improved OS. Similar findings can be found with adenocarcinoma BC. Among patients with the sarcomatoid subtype BC, by comparison, the receipt of LND and a higher LNY are not correlated with measurable improvements in OS. Ultimately, our results underscore distinct differences across subtypes that warrant individualized investigation to aid in personalized surgical decision-making for patients.

This stark divergence with respect to the benefits of LND likely reflects underlying biological differences for the different subtypes. Variants of BC have been long described to have distinct clinical behaviors as well as pathophysiologies compared with UC [16]. An analysis of 528 VBC patients from multiple institutions found recurrences happened more frequently (10-year recurrence-free survival of 30% vs. 51%, *p* < 0.001) and sooner (88 vs. 123 months, *p* < 0.01, median recurrence-free survival) when compared with UC [17]. Additionally, the patterns of recurrence are distinct for VBC. Intra-abdominal recurrence was found in 68% while only 5.8% recurred in the lung or bone. Moreover, VBC patients with non-muscle invasive diseases experienced significantly higher rates of upstaging at the time of surgery (73.8% vs. 52.4%, *p* = 0.024) and higher rates of node positivity (40.5% vs. 21.4%, *p* = 0.039) for VBC vs. UC, respectively, reflecting not just a more aggressive UC, but a distinct natural history of metastasis [6,18,19,20]. These differences extend to the molecular underpinnings of the VBC subtypes, with multiple studies demonstrating important differences in driver genes and the expression of key therapeutic molecular targets [6,21,22,23,24,25,26,27]. Thus, a surgical approach tailored to the biology of each variant warrants consideration.

Lymph node dissection carries risks to patients. Retrospective series have demonstrated increased operative time, blood loss, lymphoceles, thromboembolic events, and injury to structures like the ureter, nerves, or major vessels of the pelvis [9]. The recently completed SWOG 1011 study further reinforced the risks associated with LND. In a comparison of a standard vs. extended nodal dissection for patients undergoing radical cystectomy, grade 3 or 4 adverse events were significantly higher in the extended LND arm (16% vs. 8%), and death within 90 days was almost double (16 vs. 9 events), while no evidence of oncologic benefit was detected [3]. These findings, along with a myriad retrospective studies, highlight the added risk of this routine procedure. Thus, the decision to perform LND should be undertaken in the context of a thoughtful risk versus benefit analysis, and not by default or solely as part of a ‘maximal resection’ paradigm, informed by the biology of the disease.

The use of NAC has been suggested to affect the therapeutic benefits of LND in UC [28,29]. In an exploratory analysis of this question among VBC, our findings suggest that, here too, the impact of LND is attenuated by the receipt of NAC. For example, we identified an OS benefit associated with LND for patients with adenocarcinoma BC that did not receive NAC (NAC 83.7 months vs. 52.0 months *p* = 0.041), while those that received chemotherapy prior to surgery did not have an associated improvement in OS with LND (70.3 months vs. 68.2 months, *p* = 0.341). Conversely, the squamous subtype had preserved improvements in OS associated with LND, irrespective of NAC receipt. Variant subtypes of BC have been found to be more chemo-resistant than standard UC; thus, the perceived therapeutic benefit of NAC to any micrometastatic disease may be limited, and the risks of the delay of surgery may be heightened. Moreover, these results add to the observations that NAC attenuates LND benefits and may further play a role in patient-specific decision-making.

This study has several important limitations. The retrospective design and use of coded national clinical data resulted in inherent biases, although the concentration of these cases in high volume centers may suggest the need of large scale multi-institutional studies in the future [30]. Specifically, the unmeasured confounding of physician judgment regarding the aggressiveness of the patient’s disease as well as patient fitness or palliative intent surgeries can obscure the results. Disease-specific survival is not available in the NCDB, and there are health related factors that may impact the decision to perform a LND or receive NAC and lead to competing risks of overall survival. Coding errors may also occur and further limit the over-reliance of retrospective national data. These findings are hypothesis generating, and only randomized clinical trial can establish a causal link between nodal dissection and the oncologic outcomes in VBC. We do not suggest here that surgeons should omit LND during RC for certain VBC subtypes, rather that we find differential therapeutic benefits across groups which warrant further study and may inform individual decision-making based on risk–benefit analyses. Our findings provide retrospective support for use of LND in the squamous and adenocarcinoma subtypes of bladder cancer but could not find a correlation with improved overall survival in the setting of other variants. Ultimately, these results underscore distinct differences across subtypes that warrant individualized investigation to aid in personalized surgical decision-making for patients.

## 5. Conclusions

The role of LND across VBC subtypes is not clearly defined and warrants further investigation to develop a more risk-adaptive approach. We demonstrate heterogeneity with respect to the OS benefit associated with LND at the time of surgery. Among certain VBC subtypes, LND may not offer significant therapeutic benefits, while LND in squamous and adenocarcinoma VBCs is correlated with improved survival.

## Figures and Tables

**Table 1:** Demographic and clinical description of total variant histology cohort.

	Entire Cohort *LND*	Entire Cohort *No LND*	*p-Value*	*Micropapillary (n = 426)*	*Sarcomatoid* *(n = 704)*	*Squamous* *(n = 28,491)*	*Adenocarcinoma* *(n = 601)*	*Neuroendocrine* *(n = 614)*	*Plasmacytoid* *(n = 75)*
Age	70	71	0.73	70.9	72.9	71.64	67.83	72.61	68.98
Sex			0.69						
Male	74.8%	73.2%		77.0%	66.4%	75.4%	63.4%	77.7%	73.2%
Female	25.2%	26.8%		23.0%	33.6%	24.6%	36.6%	22.3%	26.8%
cT			0.34						
T0	0.7%	1.0%		0.1%	0.4%	0.6%	0.8%	0.3%	1.1%
T1	35.6%	34.6%		35.4%	21.6%	52.8%	29.7%	15.7%	21.1%
T2	49.7%	46.6%		50.8%	55.8%	36.1%	40.4%	62.4%	50.1%
T3	8.5%	9.7%		7.4%	11.9%	4.7%	12.4%	10.3%	12.2%
T4	5.5%	8.1%		6.2%	10.2%	5.7%	16.7%	11.2%	15.6%
cN			<0.001						
c0	78.4%	61.9%		79.3%	77.5%	75.3%	70.5%	70.3%	85.2%
c1	2.8%	2.2%		4.7%	3.7%	1.7%	3.7%	5.3%	1.6%
c2	2.4%	1.9%		3.6%	2.0%	1.6%	2.7%	4.8%	1.6%
c3	0.5%	0.3%		1.4%	0.4%	0.4%	0.1%	0.4%	3.3%
cX	15.8%	18.4%		10.9%	16.3%	21.1%	22.8%	19.2%	8.2%
pT			<0.001						
T0	6%	7.1%		4.6%	5.2%	3.4%	2.5%	6.8%	11.2%
T1	10.1%	4.4%		14.3%	7.9%	29.4%	14.4%	6.4%	1.6%
T2	28.3%	31.5%		25.6%	31.2%	25.4%	28.9%	41.6%	12.5%
T3	36.0%	41.0%		35.0%	38.5%	16.4%	34.7%	33.6%	31.7%
T4	19.6%	16.0%		19.2%	17.2%	8.4%	19.5%	11.6%	43.1%
NAC			0.93						
Yes	20.4%	18.3%		18.9%	13.3%	21.4%	13.8%	36.5%	13.3%
No	79.6%	81.7%		81.1%	86.7%	78.6%	86.2%	63.5%	86.7%
pN status			<0.001						
pN0	73%	0%		31.5%	43.0%	27.4%	39.8%	32.1%	45.3%
pN+	27%	0%		35.4%	14.8%	4.2%	11.3%	11.7%	32.0%
pNx	0%	100%		33.1%	42.2%	68.4%	48.9%	56.2%	22.7%

**Table 2:** Multivariable Cox Proportional Hazards model across variant subtypes.

	Micropapillary	*p*	Sarcomatoid	*p*	Squamous	*p*	Adenocarcinoma	*p*	Neuroendocrine	*p*
AGE	1.05 (1.03–1.08)	<0.001	1.01 (1.00–1.03)	0.04	1.02 (1.02–1.03)	<0.001	1.02 (1.01–1.03)	<0.001	1.03 (1.02–1.06)	<0.001
CT STAGE	1.23 (0.84–1.59)	0.56	1.48 (1.19–2.69)	0.77	1.21 (1.13–1.40)	<0.001	1.32 (0.54–2.71)	0.93	1.56 (1.21–1.96)	0.003
NAC	0.98 (0.63–1.5)	0.91	1.38 (0.990–1.980)	0.06	0.87 (0.59–1.29)	0.31	1.18 (0.83–1.60)	0.613	0.09 (0.07–0.94)	0.002
LN Y/N	1.73 (0.88–3.43)	0.17	1.06 (0.75–1.50)	0.70	0.50 (0.44–0.58)	<0.001	0.065 (0.045–0.93)	0.030	0.76 (0.04–1.50)	0.120
FACILITY TYPE Non-Academic (Reference) ACADEMIC	1.13 (0.83–1.32)	0.38	1.23 (0.67–1.63)	0.53	1.04 (0.83–1.25)	0.67	1.35 (0.93–1.74)	0.25	0.98 (0.74–1.42)	0.68
CN+ STATUS	1.21 (0.93–1.48)	0.19	1.32 (0.84–1.69)	0.39	1.66 (1.34–1.83)	0.03	1.08 (0.73–1.43)	0.41	1.34 (1.13–1.50)	0.02
INSURANCE STATUS PRIVATE (REFERENCE) MEDICARE/MEDICAID	1.05 (0.67–1.54)	0.67	0.95 (0.43–1.87)	0.84	1.13 (0.84–1.28)	0.19	1.04 (0.83–1.43)	0.83	1.31 (0.65–1.95	0.89

## Data Availability

The original data presented in the study are openly available in the publicly available NCDB.

## Supplementary Materials

The following supporting information can be downloaded at: https://www.mdpi.com/article/10.3390/cancers17152536/s1, Supplementary Table S1—Median overall survival between patients that received LND and those that did not, separated by NAC status (months). Supplementary Figure S1. Kaplan–Meier Analysis stratified by nodal yield.
