# Supplementary material for: Evaluating the Therapeutic Role of Lymph Node Dissection in Variant Subtype Bladder Cancer
Source: Cancers (Basel). 2025 Jul 31;17(15):2536. doi: 10.3390/cancers17152536 (PMC12346734; doi:10.3390/cancers17152536)
Supplement: Supplementary file 1 [file cancers-17-02536-s001.zip › cancers-3710443-supplementary.pdf]

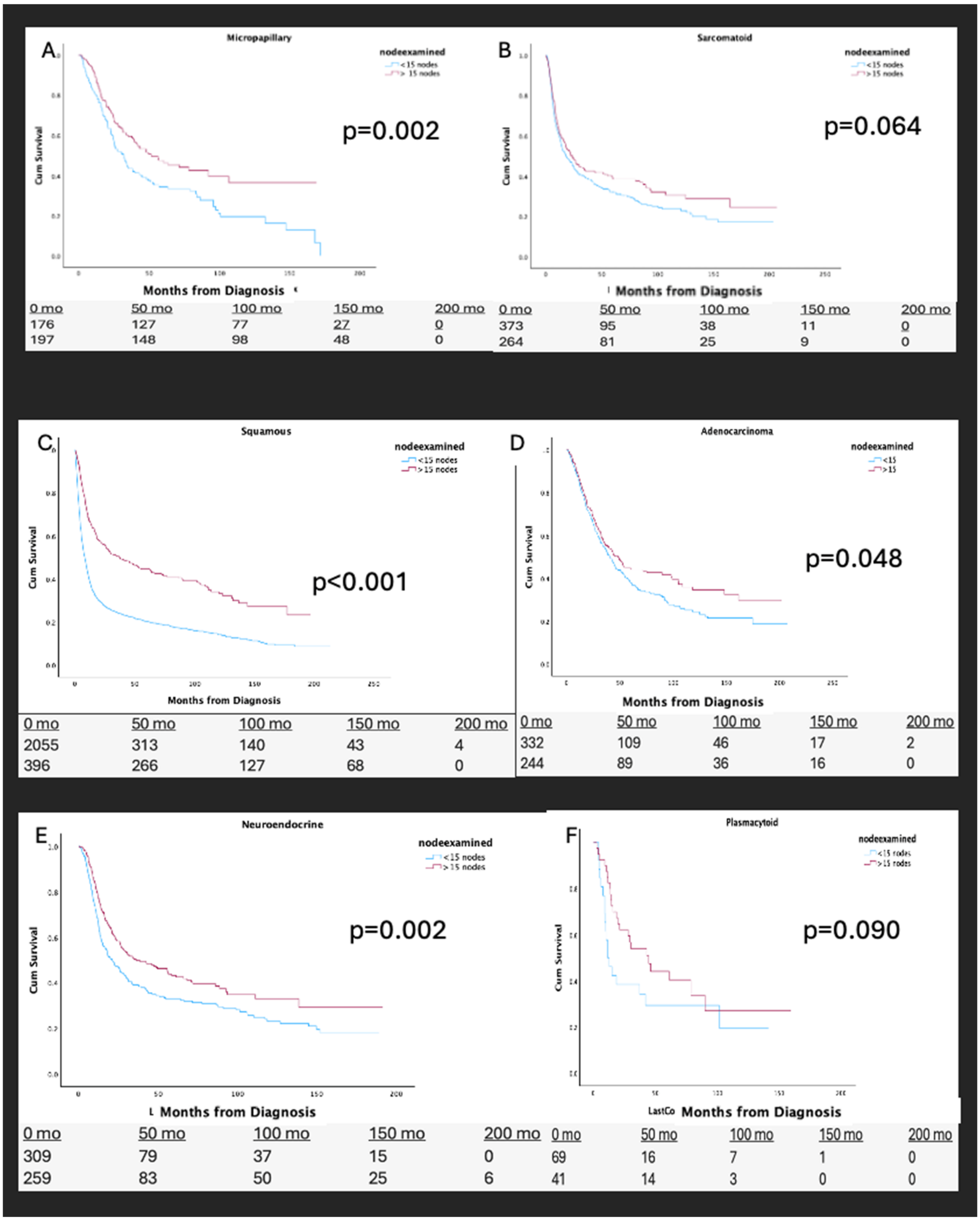

Supplementary Figure S1. Kaplan–Meier analysis stratified by nodal yield

Supplementary Table S1 – Median overall survival between patients that received LND and those that did not, separated by NAC status (months).

	NO NAC RECEIVED			NAC RECEIVED		
VARIANT	LND	No LND	p value	LND	No LND	p value
MICROPAPILLARY	69.0	64.6	0.673	61.8	34.2	0.184
SARCOMATOID	51.2	42.6	0.840	73.2	18.1	0.174
SQUAMOUS	98.4	75.9	<0.001	83.6	54.6	<0.001
ADENOCARCINOMA	83.7	52.0	0.041	70.3	68.2	0.341
